# FOXP3 Subcellular Localization Predicts Recurrence in Oral Squamous Cell Carcinoma

**DOI:** 10.1371/journal.pone.0071908

**Published:** 2013-08-20

**Authors:** Donald T. Weed, Gail Walker, Adriana C. De La Fuente, Ronen Nazarian, Jennifer L. Vella, Carmen R. Gomez-Fernandez, Paolo Serafini

**Affiliations:** 1 Department of Otolaryngology, University of Miami, Miller School of Medicine, Miami, Florida, United States of America; 2 Biostatistics and Bioinformatics Core, University of Miami, Miller School of Medicine, Miami, Florida, United States of America; 3 Department of Microbiology and Immunology, University of Miami Miller School of Medicine, Miami, Florida, United States of America; 4 Department of Pathology, University of Miami, Miller School of Medicine, Miami, Florida, United States of America; 5 Sylvester Comprehensive Cancer Center, University of Miami, Miami, Florida, United States of America; The University of Hong Kong, Hong Kong

## Abstract

Forkhead box protein P3 (FOXP3) expression in tumor infiltrating CD4^+^T cells is generally associated with an intrinsic capacity to suppress tumor immunity. Based on this notion, different studies have evaluated the prognostic value of this maker in cancer but contradictory results have been found. Indeed, even within the same cancer population, the presence of CD4^+^FOXP3^+^T cells has been associated,with either a poor or a good prognosis, or no correlation has beenfound. Here, we demonstrate,in patients with oral squamous cell carcinoma (OSCC), that what really represents a prognostic parameter is not the overall expression of FOXP3 but its intracellular localization.While overallFOXP3 expression in tumor infiltrating CD4^+^T cells does not correlate with tumor recurrence, its intracellular localization within the CD4 cells does: nuclear FOXP3 (nFOXP3) is associated with tumor recurrence within 3 years, while cytoplasmicFOXP3 (cFOXP3) is associated with a lower likelihood of recurrence. Thus, we propose elevated levels of the cFOXP3/nFOXP3 ratio within tumor infiltrating CD4^+^ T cells as a predictor of OSCC recurrence.

## Introduction

Tumor infiltrating immune cells are an important component of the tumor microenvironment and are thought to actively participate intumor progression. While the infiltration of effector lymphocytesisgenerally associated with a good prognosis, the infiltration of other immune cell populations (i.e.Myeloid Derived Suppressor Cells (MDSC) and T regulatory cells (Treg)) isthought to promote tumor progression by restraining tumor immunity and promoting neoplastic cell invasion and metastasis [1]. The identification of FOXP3, CD25 and CD4 as Treg associated markers prompted the investigation of the presence of this population in the immune infiltrate as a prognostic marker in various human malignancies [2]. FOXP3, in particular, has been widely used as a single marker to evaluate the prognostic value of tumor infiltrating Treg. Despite the initial enthusiasm [3], contradictory results were obtained. Some studies showed that tumor-infiltrating FOXP3^+^ T cells have been associated with poor prognosis, consistent with the initial hypothesis that FOXP3^+^Treg inhibit antitumor immunity [2], [4]; while other studies found that FOXP3^+^ T cells are associated with a favorable prognosis [2], [4]. In addition, other reportsshowed no correlation between the tumor infiltrating FOXP3 and clinical outcome [2], [4]. Contradictory reportsalso arise from studies restricted topatients with only oral cavity carcinoma, suggesting that these discrepancies are not caused by differences in the biology of the various malignancies analyzed. Indeed, while initial studies associate the tumor infiltration of FOXP3^+^T cellswith a worse prognosis [5], [6], other reports associate the infiltration of FOXP3^+^T cells with a better survival [7] or with better locoregional control of the tumor [8]. No significant associations were found in other studies [9]. Although technical differences in Treg quantification (i.e.different antibody clones used, scoring system, number of associated markers considered) may explain these contradictory reports, the role of biological components also needs to be considered. Indeed, it is known that, contrary to murine Treg, human T cells may transiently express FOXP3 upon activation [10]. In this case, FOXP3 expression is not indicative of a regulatory function but, instead, of either incompletely activated effector cells [11] or activated memory effector T cells [12]. Thus, although the effect of FOXP3 onactivated T cellsmay down-regulate some of theireffector functions, its expression could identify two distinct subsets of tumor infiltrating lymphocytes with opposite effects on tumor outcome. An important breakthrough can derive from the work ofMagg et al. [13] demonstrating that activated human effector T cells express FOXP3 mainly in the cytoplasm whereas Tregare characterized mostly by a nuclear localization of this important transcription factor [13].

In this retrospective case-control study, we examined the prognostic value of FOXP3 with respect to recurrence of OSCC taking into account the subcellular localization of FOXP3 within CD4^+^tumor infiltrating cells. The results indicate that the presence of CD4^+^ cells expressing FOXP3 in the cytoplasm is associated with a favorable prognosis whereas its nuclear localization correlates with an increasedrisk of recurrence. In light of these results,we propose the use of cFOXP3/nFOXP3 ratio as a prognostic factor in OSCC.

## Materials and Methods

This study was approved by the University of Miami IRB before initiation.Due to the retrospective nature of the study and the lack of personal identifier in the specimens evaluated the requirement for informed consent was waived by the IRB.

### Patients and Specimen Selection

We selected specimens from patients who underwent glossectomy (with or without neck dissection) and without prior treatment by either radiation or chemotherapy. Subjects were identified from among those treated at our tertiary referral academic medical center between 1/1/2001 and 12/31/2010 by search of a registry of CPT (Current Procedural Terminology) codes for glossectomy (41120 glossectomy less than one-half of tongue, 41130 hemiglossectomy, 41135 partial glossectomy with unilateral neck dissection). Only patients with a diagnosis of SCC of the oral tongue staged T1 or T2 (all summary stages eligible) whose surgery was limited to a partial glossectomy with or without neck dissection were included. We excluded patients with a diagnosis of in-situ carcinoma, T3 or T4 disease, involvement of other oral or oropharyngealsubsites, need for any form of mandibulectomy or other more extensive surgical procedure, a prior history of radiation to the head and neck or treatment with chemotherapy, or any history of immunosuppression or immune compromise.Additional criteria required availability of sufficient residual archival paraffin embedded tissue from the surgical resection for experimental analysis and documentation of disease status three years from date of surgery. A total of 392 clinical records were obtained, with 49 meeting inclusion criteria.

### HPV Test

Patients’ HPV status was determined by the head and neck pathology core at University of Miami using standard IHC protocols to detect p16 of Human papillomavirus.

### Immune-fluorescence Staining

Four µm sections underwent deparaffinization, rehydratation, and incubation for 30 min at RT in a sodium borhydride solution (0.5 mg/mL in PBS; EMDGibbstown, NJ, USA) to reduce sample autofluorescence. Antigen retrievalwas performed by a 15 min incubation at 95°C in EDTA antigen retrieval solution pH = 9 (GIBCO Carlsbad, CA, USA). The slides were then incubated with Image-iT (Invitrogen) for 30 min at RT followed by incubation (1 h at RT) with PBS containing 1% BSA and 0.3% Triton-X100 to permeabilize the tissue and to block non-specific binding. Samples were incubated O/N at 4°C with the mouse monoclonal, anti-human FOXP3 antibody ab20034 (clone237/E7, dilution 1/25, Abcam) and the goat polyclonal anti-human CD4 antibodyAF-379-NK, (R&D bioscience, dilution 1/20) in PBS with 1% BSA. After three washes with PBS, samples were labeled for 1 h at RT with the Alexa-555 conjugated anti-mouse antibody (for FOXP3) and with the Alexa-488 conjugated anti-goat antibody (for CD4). Both antibodies were used at a 1/400 dilution in PBS/BSA (1%). Finally, sections were counterstained in PBS containg 2 mMDAPI (Invitrogen),for 15 min at RT, rinsed with PBS and coverslips were mounted using Biomeda gel mounting media (Electron Microscopy sciences, Hatfield, PA, USA). Slides were analyzed with ZeissAxiovert Microscope and the Zeiss Axiovision LE software (Zeiss, Thornwood, NY) at 20×magnification or with the SP5 spectral confocal inverted microscope (Leica) at the University of Miami imaging core facility.

### Image Selection, Processing and Quantification

Areas of interest corresponding to the neoplastic lesion of the tissue were identified by an experienced pathologist in serial section stained with H&E. Microphotographs of 5 random fields at 20X were taken in the corresponding area of the fluorescence labeled sections with a Zeiss Axiovert Microscope. Images for each patient were qualitatively evaluated with ImageJ (http://rsbweb.nih.gov/ij/ [14]) and processed together by cell-profiler (www.cellprofiler.com [15]–[19]) for image cytometer analysis. Briefly, TIF images were split into the separate red, blue and green channels, and each image was converted to a gray scale. Nuclei were identified as the primary object using the blue (DAPI) channel setting the diameter of the nuclei between 20 and 50 pixels, discarding the object touching the border of the image, and using the Kapur Global threshold method with a correction factor of 0.5 and the lower and upper bounds on threshold 0.3–0.9. Clumped objects were distinguished by shape and the size of the smoothing filter was automatically calculated. Local maxima that were closer than 20 pixels were automatically suppressed. Once the nuclei were identified, cells were identified as secondary objects using the autofluorescence in the green channel. Starting from the nuclei a propagation method using the Otzu Global two classes thresholding method was used. 1 was used as threshold correction factor (0.0–1.0 range) and 0.05 as regularization factor. Finally, the cytoplasm as tertiary object was identified as the area included in the cells (secondary object) but not in the nuclei (primary object). For each cell, mean intensity of the nuclei (primary object) and of the cytoplasm (tertiary object) in the red (FOXP3) and in the green (CD4) channels were exported into an excel spreadsheet. Median and interquartile range (defined as 2×Q3-median) calculated for CD4 and FOXP3 intensity (in the nucleus and in the cytoplasm) were used to calculate the threshold empirically defined as: median+(1.7×interquartile range). Cells whose mean intensity signal was superior to the threshold were counted as positive.

### Statistical Analysis

Exploratory analysis of FOXP3 expression included summary statistics, normality tests and plots by case-control status. Relationships between markers were examined with Spearman and Pearson correlation, and the Wilcoxon rank sum test was used to compare marker levels of cases versus controls. Univariate logistic regression was used to test markerassociation with recurrence. Comparison of markers with respect totheir prognostic valuewas based ondifferences in the area under the empirical receiver operating curves (ROC; one minus specificity vs sensitivity). Consideration of multivariate models was limited to testing the addition of baseline patient characteristics to the single marker model with the best (highest) AUC. All statistical analysis was performed in SAS® v 9.3.

## Results and Discussion

### Study Design

Different hypotheses have been proposed to explain the inconsistent results obtained by different studies on the predictive role of tumor infiltrating FOXP3^+^ CD4^+^ cells in HNSCC [20]. For example, a non-homogeneous patient population could have been selected, non-optimal antibody clones may have been used, or semi-quantitative and subjective enumeration methods may have been used. To overcome these possible technical pitfalls we designed a retrospective study of a homogenous cohort of patients with T1 orT2 oral tongue squamous cell carcinoma.The rationale for inclusion of early T stage patients regardless of summary stage was to capture a population of patients whose tumor size and site were homogeneous but whose tumors exhibited sufficient biological variability with regard to clinical aggressiveness (as measured by presence or absence of regional metastasis, as well as variations in primary site clinicopathologic predictors such as depth of invasion, perinueral invasion, and lymphovascular invasion) to anticipate a likelihood of recurrence events sufficient for experimental analysis. Out of 392 clinical records evaluated, specimens from 49 patients were included in the studyandthen classified as cases or controls based on whether or notmedical records includedevidence of disease recurrence (local, regional, distant or combination thereof) within 36 months after surgery (allowing a 2.5 month buffer). This resulted in 19 cases of recurrence at a median time of 12.3 months, (range 3.7–38.5),and 30 non-recurrent controls with a median follow up of 59.7 months (range 36.9–103). We note that where additional follow up information was available for controls, there was no indication of disease recurrenceafter three years. Analysis ofclinical parameters revealed that tumor recurrence was associated significantly with the use of tobacco and alcohol (p = 0.031, table 1), and marginally with patient age (p = 0.074), while surprisingly no significant correlationswere found with race, gender, T stage, TNM stage, or clinicopathologic tumor characteristics (table 1). HPV-p16 analysis by immunohistochemistry reveals that all the patients were HPV negative (data not shown) confirming the previous data indicating a low HPV frequency in oral tongue tumors [21], [22]. Thus, no association with HPV status can be made.

**Table 1:** Patient characteristics and association with recurrence.

	Patients		Risk of recurrence	
	N	%	OR (95% CI)	p-value
Age at surgery (years)	26	53.1		
34 to 63 (median)			reference	
64 to 93	23	46.9	3.0 (0.9–9.8)	0.074
Gender				
Male	28	57.1	reference	
Female	21	42.9	1.9 (0.6–6.2)	0.274
Race				
White, non Hispanic	32	66.7	reference	
Hispanic	16	33.3	0.4 (0.1–1.4)	0.151
Unknown	1	−		
Alcohol & tobacco use				
Neither	14	29.8	reference	
Alcohol only	4	8.5	3.7 (0.4–38.0)	0.276
Tobacco only	11	23.4	0.8 (0.1–6.0)	0.841
Both	18	38.3	5.8 (1.2–28.3)	0.031
Unknown	2	−		
T-stage				
T-1	34	69.4	reference	
T-2	15	30.6	2.4 (0.2–8.3)	0.170
Pathologic grade				
Well differentiated	23	48.9	reference	
Moderately (20) orpoorly (4) differentiated	24	51.1	2.3 (0.7–7.6)	0.175
Unknown	2	−		

N: number patients. OR: Odds ratio estimate for the risk of recurrence comparing patient groups shown in left hand column. CI: Confidence interval. *p-value:* Wald test.

### FoxP3 Expression does not Correlate with the Clinical Outcome

To evaluate whether tumor infiltrating Treg can serve as a prognostic marker in this defined patient cohort, paraffin embedded sections of the tumor were stained with hematoxylin and eosin (to confirm the diagnosis and better localize the tumor) and serial sections labeled with antibodies specific for human CD4 and human FOXP3 and counterstained with DAPI.

The anti-human FOXP3 antibody clone 236A/E7 was selected and used because it targets an epitope between aminoacid 105 and 235 of human FOXP3 [23] and, thus, contrary to other clones, can recognize the full length FOXP3, the isoform lacking exon 2 (aa 70–105, FOXP3Δ2) [24] and the FOXP3Δ2Δ7 [24], [25] lacking exons 2 and 7 (aa 239–260). Furthermore, comparative studies using different anti-human FOXP3 antibodies confirmedthe capacity of this clone to give more consistent results in flow cytometry [26] and to be more suitable for the staining of paraffin embedded tissue [27].

**Figure 1:**
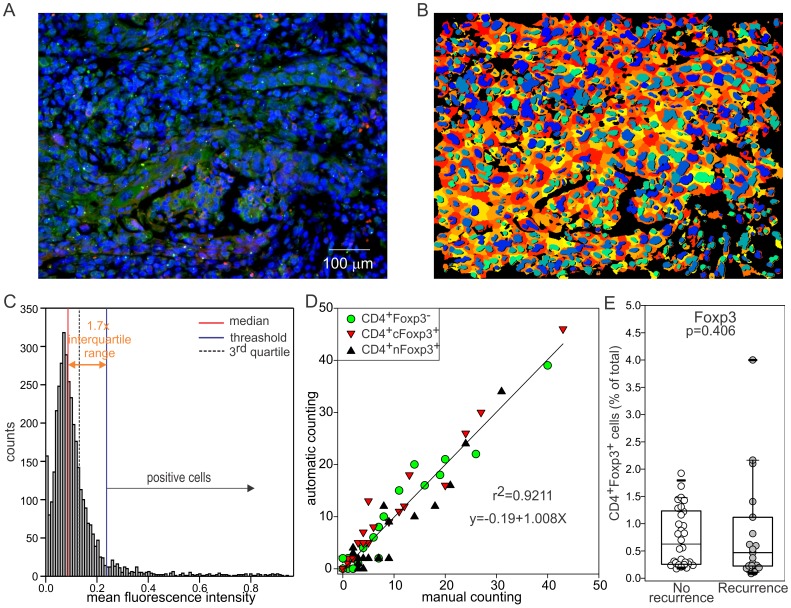
A computer assisted method for image quantification. A) Immune fluorescence images 20X were taken on slides labeled with anti-CD4 (green) and anti-FOXP3 (red) antibodies and counterstained with DAPI for the nuclei. Images were segmented using Cellprofiler using the setting optimized to correctly segment lymphocytes as described in the material and methods. B) processedpicture showing the nuclei in a blue scale and the cytoplasm in red/yellow gradient. As shown in C), MFI values assume a “quasi” Gaussian distribution close to the 0. Empirically we found that a cell is considered positive by different investigators when its MFI is greater than a threshold (blue line) defined as: median (MFI)+1.7×(2XQ3-median). D) This method and this threshold were validated by counting CD4^+^Foxp3^-^ (green circle), CD4^+^nFOXP3^+^ cells (black triangle) or CD4^+^cFOXP3^+^ (red triangle) cells in 20 pictures and by correlating those counts with the one obtained from the computer. The reported R^2^ and equation correspond to the interpolated line when all the 3 cell types are included in the analysis. When CD4^+^FOXP3^-^, CD4^+^cFOXP3^+^ or CD4^+^nFOXP3^+^ are counted the R^2^ values are 0.942, 0.956, and 0.890 respectively while the slope values are 0.983, 1.019, and 0.999 indicating a good correspondence between the “automatic” and the manual counting. E) CD4+FOXP3+ (regardless of the localization) were evaluated in recurrent or tumor free patients.

Since the visual enumeration of positive cells presents intrinsic limits even in trained investigators (i.e. researcher subjectivity, the incapacity of the human eyes to discriminate linearly differences in signal intensity, the environmental light conditions, the adaptation to the signal intensity of the operator eyes, etc) that could limit the power of theanalysis, CD4^+^FOXP3^+^ cells were quantified using a computer-aided method for image analysis (Fig. 1). Briefly, immune fluorescence microphotographs were taken in the tumor area, images were segmented (Fig. 1B) and the signal intensity for CD4 and FOXP3 werequantified in each individual cellas described in the materials and methods. Thedistribution of the mean fluorescence intensityvalues appear to assume a “quasi” Gaussian distribution close to the 0 that is in accordance with the fact that in each specimen most of the cells are negative for either CD4 or FOXP3 (Fig. 1C), Empirically we found that a cell is considered positive by different investigators when its MFI is greater than a threshold (blue line) defined as: median (MFI)+1.7×(interquartile range) as demonstrated by the strong correlation between “manual” and computer based counting (Fig. 1D).

The percentage of CD4^+^FOXP3^+^ cells was evaluated with this method for each patient as well as thedifferences between patients with or without recurrence.Despite the use of a homogenous population, validated antibodies, and a computer aided method for tumor infiltrating Treg enumeration, no association was found between the percentage of CD4^+^FOXP3^+^ within the and tumor recurrence (p = 0.406, Fig. 1 E).

### FOXP3 Subcellular Localization within the CD4^+^cells Predicts Tumor Recurrence

Considering the importance that the immune system plays in HNSCC outcome and the crucial role that Tregcells play in restraining tumor immunity [20], the finding that FOXP3 expression within the CD4^+^T cells is not associated with the clinical outcome was quite intriguing.

**Figure 2:**
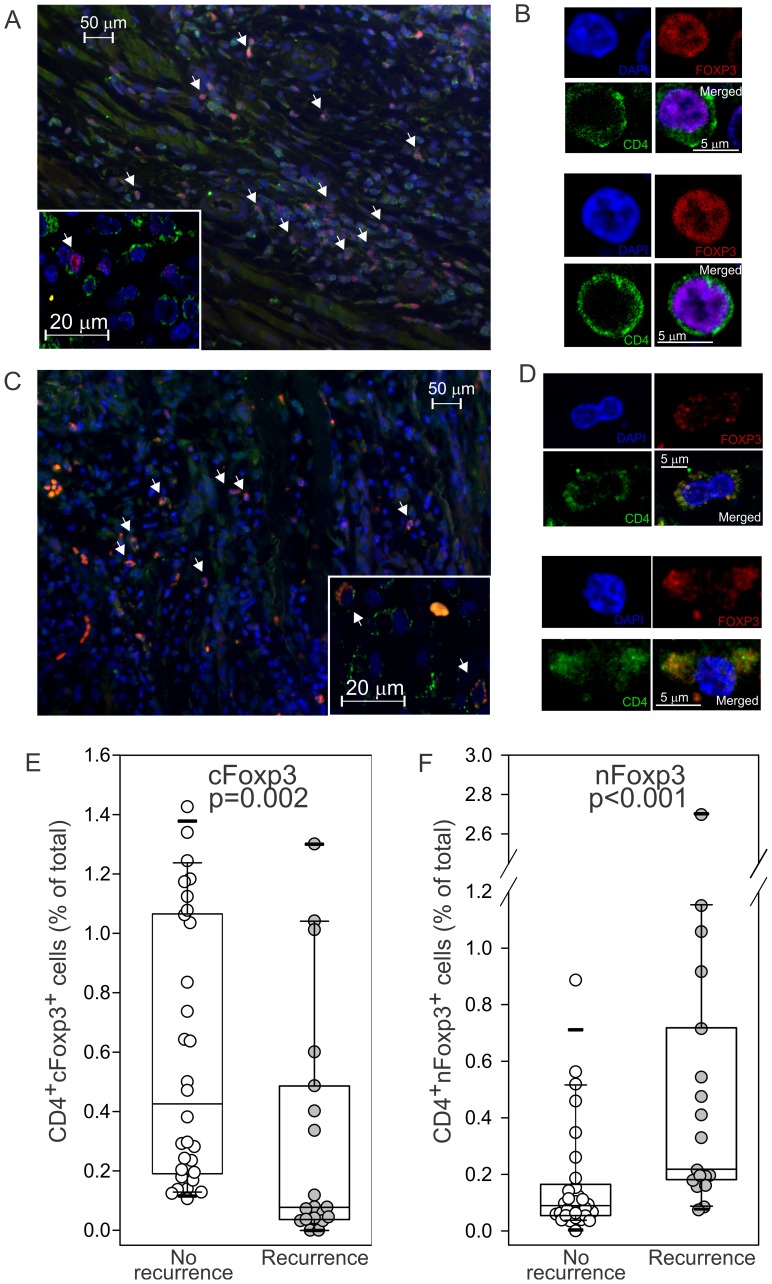
FOXP3 subcellular localization is predictive of tumor recurrence. Tumor specimens werelabeled with anti-CD4 (green) and anti-FOXP3 (red) antibody and counterstained with DAPI (blue). Representative immune fluorescence microscopy photograph from (**A**) recurrent or (**C**) tumor free patients are shown. The corresponding images from the confocal microscope are shown in the inner panel. Different localization confirmed by confocal microscopy and examples of nuclear (**B**) or cytoplasmic (**D**) localization are shown. Images were analyzed by cell profiler and the percentage of CD4^+^cells positive for FOXP3 only in the cytoplasm (cFOXP3), or only in the nucleus (nFOXP3), are reported respectively in **E**and **F**. Wilcoxon rank-sum test *p* value is reported.

Nevertheless, it is important to note that contrary to the mouse model, FOXP3 in the human can be expressed not only by Treg but also by activated T cells [28]. Moreover, it was recently reported that while in regulatory T cells FOXP3 expression is confined primarily to the nucleus, in activated T cells it is localized primarilyin the cytoplasm of CD4^+^ T cells [13]. In accordance with this observation, qualitative evaluation of the immune-fluorescence section revealed the presence of two subsets of tumor infiltrating FOXP3^+^CD4^+^ T cells: T cells characterized by a nuclearFOXP3 expression (Fig. 2 A–B) and T cells whoseFOXP3 expression was localized in the cytoplasm (Fig. 2C–D). These data, confirmed by confocal microscopy analysis (Fig. 2B–C and A–B insert), could suggest that both activated effector T cells and regulatory T cells infiltrating the tumor may express FOXP3.

Since functional analyses were not feasible due to the nature of the experimental sample (paraffin embedded), we evaluated whether the subcellular localization of FOXP3 within the CD4^+^ T cells could be predictive of recurrence in our cohort of OSCC patients. Briefly, cytoplasmic and nuclear regions were defined with Cellprofiler using DAPI for the nuclear staining and green channel autofluorescence to identify the cellular membrane. Signal intensity in the FOXP3 red channel was then measured in the cytoplasm and in the nucleus and cells were considered positive when signal intensity was higher than the threshold empirically defined asmedian +1.7 times the interquartile range. Surprisingly, FOXP3 expression in both cellular compartments was mutually exclusive (data not shown),further suggesting that two well defined subsets of FOXP3^+^CD4^+^T cells infiltrate the tumor. Furthermore, while the percentage of cytoplasmicFOXP3^+^T cells was associated with a favorable prognosis (p = 0.002, Fig. 2F), the presence of nuclear FOXP3^+^CD4^+^T cells (nFOXP3) within the CD4 significantly correlated with tumor recurrence (p<0.001, Fig. 2G).

### The Ratio between the Nuclear and Cytoplasmicfoxp3 is the Most Reliable Prognostic Factor in OSCC

**Figure 3:**
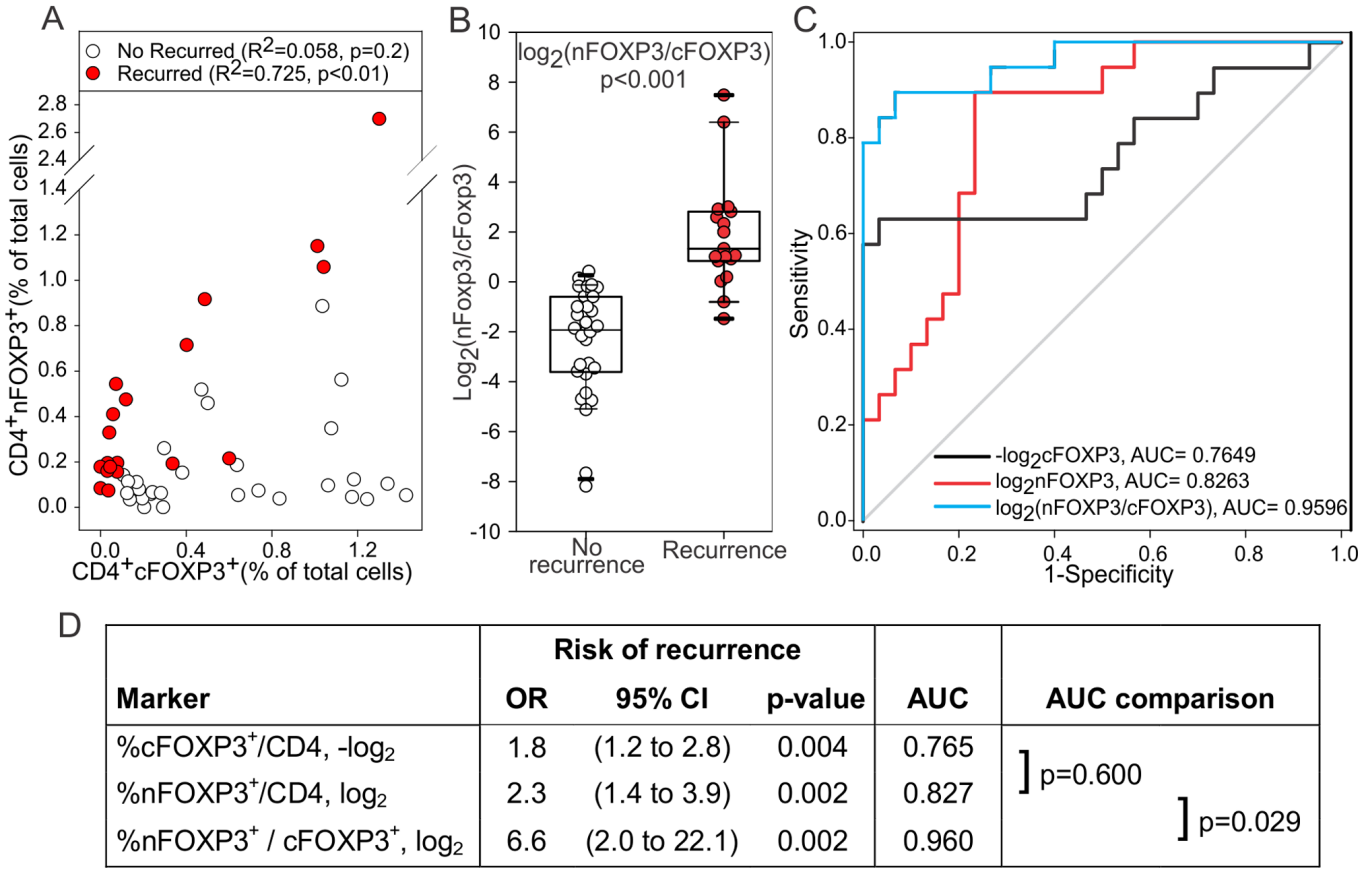
nFOXP3/cFOXP3 ratio is a reliable indicator of tumor recurrence in OSCC. A) The percentage of CD4^+^cFOXP3^+^cells was plotted against the percentage of nFOXP3^+^cells in tumor free (white circle) or recurrent (red circle) patients. Linear correlation between the two parameters was evaluated. B) Data from the percentage of FOXP3 positive cells in the nucleus or in the cytoplasm were transformed by adding 0.001 to 0 values and taking the ratioof nuclear to cytoplasmicFOXP3^+^CD4^+^ tumor infiltrating T cells. Results are depicted after log_2_ transformation with Wilcoxon rank sum test *p*-value. C) ROC plots. D) Univariate logistic regression and ROC analysis. **OR**: Odds ratio estimate for the increased risk of recurrence per twofold increase (log_2_) or 50% decrease (−log_2_) in marker shown in left hand column. **CI**: Confidence interval. **AUC**: area under the empirical receiver operating curve (ROC) shown in upper right panel.

*In silico* models of the interaction between regulatory and effector T cells [29] predict that an equilibrium should be reached between the concentration of effector and regulatory T cells. This prediction seems to be confirmed by some of our preclinical observations in the A20HA lymphoma model in which, during the instauration of tumor induced T cell tolerance, the number of Tregcells specific for a tumor antigen matched the number of effector T cells with the same specificity [30](and Serafini unpublished data). Interestingly, and in accordance with the proposed models, while data obtained from the 30 recurrence free controlsshowed no correlationbetween nuclear and cytoplasmic FOXP3^+^CD4^+^T cells (p = 0.198 Pearson; p = 0.499 Spearman), there was a significant positive linear correlationwhen in the 19 cases of diseaserecurrence (r^2^ = 0.725; p<0.001). This analysis indicated a 1.37-fold increase in Treg cells per unit increase in %cFOXP3^+^/CD4^+^ cells (Fig. 3A).

The relationship between Treg and effector FOXP3^+^ can also be seen by considering the ratio nFOXP3^+^/cFOXP3^+^which showed a significant difference (p<0.001) between cases and controls (Fig. 3B).

Univariate logistic regression was used to further study nuclear FOXP3, cytoplasmicFOXP3 and the ratio of these two markers in relation to recurrence. Data transformations were applied to obtain odds ratio estimates of the increased risk of recurrence per twofold increase (log_2_(nFOXP3) and log_2_(ratio nFOXP3/cFOXP3) ) or 50% decrease (−log_2_(cFOXP3)) in the marker. Receiver operating curves (ROC) and corresponding area under the curve (AUC) are shown in Fig. 3C. AUC increased from 0.765 for cytoplasmic FOXP3 to 0.827 for nuclear FOXP3 but the difference was not statistically significant (p = 0.600). The ratio of nuclear to cytoplasmic FOXP3 resulted in an AUC of 0.960, which was a significant increase in comparison to nuclear FOXP3 (p = 0.029) (Fig. 3D). Since the ratio of nuclear to cytoplasmicFOXP3^+^ CD4^+^ cells gave the highest AUC, we estimated the effect of this marker after adjustment for each of the baseline characteristics previously considered. None of these covariates were significant although T-stage was marginally significant (p = 0.097) (table 2).

**Table 2:** Prognostic effect of the ratio of nuclear to cytoplasmic FOXP3^+^CD4^+^ cells, adjusted for baseline characteristics.

	#Adjusted effect of %nFOXP3^+^/cFOXP3^+^			Covariate effect		
Model covariate	OR	95% CI	p-value	OR	95% CI	p-value
Age a surgery: ≥64 vs ≤63 years	9.2	(1.7 to 48.9)	0.009	5.4	(0.5 to 62.9)	0.178
Gender: female vs male	8.4	(2.0 to 35.5)	0.004	4.7	(0.5 to 49.2)	0.194
Race: Hispanic vs white nonHispanic	6.3	(1.9 to 20.5)	0.003	0.7	(0.1 to 6.8)	0.739
Current or former use of tobacco and alcohol:	7.2	(1.9 to 27.2)	0.004	–	–	–
Tobacco only	–	–	–	0.2	(0.01 to 5.2)	0.301
Alcohol only	–	–	–	1.0	(0.0 to 478.2)	0.989
both	–	–	–	0.3	(0.02 to 5.0)	0.429
T-stage: T-2 vs T-1	8.2	(2.2 to 30.9)	0.002	9.1	(0.7 to 124.5)	0.097
Grade: mod/poor differentiation v well differentiated	6.5	(1.9 to 21.8)	0.002	4.2	(0.5 to 35.5)	0.193

OR: Odds ratio. CI: Confidence interval. *p-value:*Wald test.

# Risk of recurrence per twofold increase in ratio of nuclear to cytoplasmic FOXP3^+^CD4^+^ cells, adjusted for covariate shown at left.

### Concluding Remarks

Since its discovery, FOXP3 expression within the tumor infiltrating CD4^+^ cells has been evaluated as a prognostic marker for different human malignancies. Nevertheless, while some studies indicate its association with a negative prognosis, others correlate its expression with a positive prognosis or show no association. Here we show that intracellular localization is an important factor to considerwhen assessing the prognostic importance of FOXP3.In oral squamous cell carcinoma,an elevated number of tumor infiltrating CD4^+^T cellsexpressingFOXP3 in the cytoplasm are indicative of a favorable prognosis (no recurrence within three years) whereas an high concentration of CD4^+^ T cells expressing nuclear FOXP3 isstrongly associated with recurrence.Furthermore, the ratio between nuclear and cytoplasmicFOXP3^+^ CD4^+^ T cells was significantly better than either localized measure as an indicator of the risk of recurrence in patients with OSCC. Thus, we suggest the exploration of this parameter in other malignancies.
